# Addition of lactic acid levels improves the accuracy of quick sequential organ failure assessment in predicting mortality in surgical patients with complicated intra-abdominal infections: a retrospective study

**DOI:** 10.1186/s13017-018-0173-6

**Published:** 2018-03-13

**Authors:** Yun Tae Jung, Jiyeon Jeon, Jung Yun Park, Myung Jun Kim, Seung Hwan Lee, Jae Gil Lee

**Affiliations:** 1 Department of Surgery, Yonsei University College of Medicine, 50-1 Yonsei-ro, Seodaemun-gu, Seoul, 03722 Republic of Korea; 2 Yonsei University College of Nursing, 50-1 Yonsei-ro, Seodaemun-gu, Seoul, 03722 Republic of Korea

**Keywords:** Retrospective studies, Lactate, Intra-abdominal infection, Mortality

## Abstract

**Background:**

The quick sequential organ failure assessment (qSOFA) alone has a poor sensitivity for predicting mortality in patients with complicated intra-abdominal infections, and plasma lactate levels have been shown to have a strong association with mortality in critically ill patients. Therefore, this study aimed to compare the performance of qSOFA with a score derived from a combination of qSOFA and serum lactate levels for predicting mortality in surgical patients with complicated intra-abdominal infections.

**Methods:**

This retrospective study was performed at a university hospital. The medical records of 457 patients who presented to the emergency department (ED) between January 2008 and December 2016 and required emergency gastrointestinal surgery for a complicated intra-abdominal infection were reviewed retrospectively. qSOFA criteria, sequential organ failure assessment (SOFA) scores, and plasma lactate levels during their ED stay were collected. We performed area under receiver operating characteristic (AUROC) curve and sensitivity analysis to compare the performance of qSOFA alone with that of a score derived from the use of a combination of the qSOFA and lactate levels for predicting patient mortality.

**Results:**

Fifty patients (10.9%) died during hospitalization. The combined qSOFA and lactate level score was superior to qSOFA alone (AUROC = 0.754 vs. 0.717, *p* = 0.039, respectively) and comparable to the full SOFA score (AUROC = 0.754 vs. 0.795, *p* = 0.127, respectively) in predicting mortality. Sensitivity and specificity of qSOFA alone were 46 and 86%, respectively, and those of the combined score were 72 and 73%, respectively (*p* < 0.001).

**Conclusion:**

A score derived from the qSOFA and serum lactate levels had better predictive performance with higher sensitivity than the qSOFA alone in predicting mortality in patients with complicated intra-abdominal infections and had a comparable predictive performance to that of the full SOFA score.

## Background

Sepsis has been a major cause of death for many decades in critically ill patients worldwide [1, 2]. Despite its high morbidity and mortality, there is no diagnostic gold standard test for sepsis. For the past decade, sepsis had been defined as a systemic inflammatory response syndrome (SIRS) by the host to an infection [3]. Owing to advances in our understanding of the pathophysiology of sepsis, a 2016 consensus conference redefined sepsis as “life-threatening organ dysfunction caused by a dysregulated host response to infection” [4].

Based on this new definition, sepsis is now diagnosed when a ≥ 2-point change occurs in the sequential organ failure assessment (SOFA) score because of an infection. However, due to the complex nature of the SOFA score, the 2016 task force introduced quick SOFA (qSOFA) for the rapid identification of patients at a high risk of mortality in the setting where all components of the SOFA score cannot be measured [4]. The qSOFA score includes (1) systolic blood pressure (SBP) ≤ 100 mmHg, (2) respiratory rate (RR) ≥ 22 breaths/min, and (3) an altered mental status (AMS). The total qSOFA score ranges from 0 to 3. Despite its simplicity, qSOFA showed better accuracy than SIRS criteria for predicting patient mortality in non-intensive care unit (ICU) settings in many studies [5–7]. However, others have questioned its utility as a quick screening tool due to its poor sensitivity [8–10].

Because of the limited sensitivity of qSOFA, we performed this study based on three assumptions. First, qSOFA alone has a poor sensitivity for predicting mortality in patients with complicated intra-abdominal infections. Second, plasma lactate levels have been shown to have a strong association with mortality in critically ill patients [11–13]. Therefore, the combination of the qSOFA score with a score based on lactate levels should have a better prognostic performance than qSOFA alone. Finally, the combination score should have a prognostic performance comparable to that of the full SOFA score. The aim of this study was to compare the performance of qSOFA with a combined score derived from qSOFA and serum lactate levels for predicting in-hospital mortality in surgical patients with complicated intra-abdominal infections.

## Methods

### Study population

We retrospectively reviewed the medical records of 1226 adult patients who underwent emergency gastrointestinal surgery for a complicated intra-abdominal infection from January 2008 to December 2016. Among this population, 511 patients were admitted via the emergency department (ED), but 51 of these patients did not have their plasma lactate level checked. Three patients with infection source inadequately controlled were also excluded because operations with inadequate source control usually end up with critical consequences regardless of their preoperative conditions. Excluding these 54 patients, 457 patients were finally included in the study.

### Data collection

Components of the qSOFA and full SOFA scores during the patients’ ED stay were collected from the electronic medical record (EMR). The qSOFA score included one point for each of the following: (1) systolic blood pressure ≤ 100 mmHg, (2) respiratory rate ≥ 22 breaths/min, and (3) altered mental status (AMS). The patient’s baseline characteristics, initial laboratory values, operation-related data, and clinical outcomes were collected from the EMR. In our ED, the initial mental status of non-traumatic patients is recorded using the alert/verbal/painful/unresponsive (AVPU) responsiveness scale. Therefore, the AVPU scale was used instead of the Glasgow Coma Scale score when calculating the central nervous system component of the full SOFA score [14, 15].

One additional point was added to the patient’s qSOFA score when their plasma lactate level was ≥ 2 mmol/L during their ED stay. This newly calculated combined score (qSOFA + lactate score) ranged from 0 to 4. Sensitivity analysis for each criterion was performed at a cutoff value of 2 for the qSOFA score, the qSOFA + lactate score, and the full SOFA score. Plasma lactate levels were included because they have been shown to be useful in predicting mortality in critically ill patients in numerous prior studies [11–13].

This study was approved by the Yonsei University Institutional Review Board (4-2017-0726), and informed consent was waived due to the retrospective design of the study.

### Statistical analysis

A Student *t* test and the Mann-Whitney *U* test were performed for continuous variables, as appropriate, and presented as the mean ± standard deviation or median (interquartile range [IQR]). Categorical variables were presented as a frequency (%) and compared using the chi-square or Fisher’s exact test, as appropriate. Sensitivity analysis, receiver operating characteristic (ROC) curve, and area under ROC curve (AUROC) with 95% confidence intervals (CI) were analyzed for each score. To compare sensitivities and specificities of each score, generalized estimating equations and the weighted least square method were used.

The findings were considered statistically significant when *p* values were less than 0.05. Statistical analysis was performed using SPSS® Statistics 23.0 (IBM Corp., Armonk, NY), SAS (version 9.4, SAS Inc., Cary, NC, USA), and R package (version 3.1.3, https://www.r-project.org/).

## Results

### Baseline characteristics

Of the 457 patients, 407 (89.1%) patients survived, and 50 (10.9%) patients died during hospitalization. Non-survivors had a higher average age (68.94 ± 15.27 vs. 62.11 ± 16.19 years, respectively; *p* = 0.005), ASA score (*p* = 0.007), and APACHE II score (28.26 ± 9.08 vs. 20.11 ± 7.86, respectively; *p* < 0.001) (Table 1).

**Table 1:** Baseline characteristics of the study population (*n* = 457)

Variables	Total population	Survivors (*n* = 407)	Non-survivors (*n* = 50)	*p* value
Age, years	62.86 ± 16.22	62.11 ± 16.19	68.94 ± 15.27	0.005
Sex, *n* (%) male/female	280 (61.3)/177 (38.7)	253 (62.2)/154 (37.8)	27 (54.0)/23 (46.0)	0.264
Body weight, kg	58.36 ± 11.58	58.38 ± 11.32	58.23 ± 13.69	0.931
BMI, kg/m^2^	21.90 ± 3.64	21.83 ± 3.54	22.47 ± 4.42	0.243
ASA score, *n* (%)				0.007
1	131 (28.7)	120 (29.5)	11 (22.0)	
2	124 (27.1)	114 (28.0)	10 (20.0)	
3	153 (33.5)	137 (33.7)	16 (32.0)	
4	45 (9.8)	34 (8.4)	11 (22.0)	
5	4 (0.9)	2 (0.5)	2 (4.0)	
APACHE II score	21.23 ± 8.50	20.11 ± 7.86	28.26 ± 9.08	< 0.001
Comorbidity, *n* (%)				
Hypertension	172 (37.6)	145 (35.6)	27 (54.0)	0.011
Diabetes	65 (14.2)	59 (14.5)	6 (12.0)	0.633
CRF	39 (8.5)	36 (8.8)	3 (6.0)	0.787
Malignancy	212 (46.4)	183 (45.0)	29 (58.0)	0.081
Diagnosis, *n* (%)				0.632
Perforation	382 (83.6)	338 (83.0)	44 (88.0)	
Strangulation	58 (12.7)	54 (13.3)	4 (8.0)	
Ischemia	17 (3.7)	15 (3.7)	2 (4.0)	
Site, *n* (%)				0.789
Stomach	111 (24.3)	100 (24.6)	11 (22.0)	
Small bowel	163 (35.7)	143 (35.1)	20 (40.0)	
Colorectal	183 (40.0)	164 (40.3)	19 (38.0)	
Laparoscopy/open, *n* (%)	71 (15.5)/386 (84.5)	69 (17.0)/338 (83.0)	2 (4.0)/48 (96.0)	0.013

*BMI* body mass index, *ASA* American Society of Anesthesiologists, *APACHE* acute physiology and chronic health evaluation, *CRF* chronic renal failure

### Components of qSOFA, lactate level, and full SOFA score

A SBP ≤ 100 mmHg and a RR ≥ 22 breaths/min were observed in 39.4 and 26.7% of the patients, respectively. Only 3.3% of the patients showed an AMS. The non-survivor group had higher qSOFA, qSOFA + lactate, and full SOFA scores (Table 2).

**Table 2:** Distribution of qSOFA criteria, lactate levels, and qSOFA, qSOFA + lactate, and SOFA scores

Variable	Total population	Survivors (*n* = 407)	Non-survivors (*n* = 50)	*p* value
SBP ≤ 100, *n* (%)	180 (39.4)	144 (35.4)	36 (72.0)	< 0.001
RR ≥ 22, *n* (%)	122 (26.7)	98 (24.1)	24 (48.0)	< 0.001
AMS, *n* (%)	15 (3.3)	11 (2.7)	4 (8.0)	0.070
Lactate, mmol/L	1.7 [1.0–3.1]	1.6 [0.9–2.8]	3.3 [1.775–4.875]	< 0.001
Lactate ≥ 2.0 mmol/L, *n* (%)	200 (43.8)	164 (40.3)	36 (72.0)	< 0.001
qSOFA, *n* (%)				< 0.001
0	234 (51.2)	223 (54.8)	11 (22.0)	
1	145 (31.7)	129 (31.7)	16 (32.0)	
2	71 (15.5)	51 (12.5)	20 (40.0)	
3	7 (1.5)	4 (1.0)	3 (6.0)	
qSOFA + lactate score, *n* (%)				< 0.001
0	163 (35.7)	158 (38.8)	5 (10.0)	
1	147 (32.2)	138 (33.9)	9 (18.0)	
2	86 (18.8)	68 (16.7)	18 (36.0)	
3	54 (11.8)	39 (9.6)	15 (30.0)	
4	7 (1.5)	4 (1.0)	3 (6.0)	
Full SOFA	1 [0–4]	1 [0–3]	5 [3–8.25]	< 0.001

*SBP* systolic blood pressure, *RR* respiratory rate, *AMS* altered mental status, *qSOFA* quick sequential organ failure assessment, *SOFA* sequential organ failure assessment

### Predictive performance of qSOFA alone

The SIRS had the lowest predictive performance among the criteria (AUROC = 0.672, 95% CI = 0.599–0.745), though it was better than that of a single plasma lactate level with a cutoff value of 2.0 mmol/L (AUROC = 0.659, 95% CI = 0.581–0.736). The qSOFA score showed a better predictive performance compared to SIRS criteria, but this difference was not statistically significant (AUROC = 0.717, 95% CI = 0.673–0.758 vs. AUROC = 0.672, 95% CI = 0.599–0.745; *p* = 0.325). Furthermore, qSOFA had the lowest sensitivity (46%), though it had the highest specificity (86%) (Table 3).

**Table 3:** Predictive performance for each diagnosis or score

Variable	Sensitivity	Specificity	AUROC
Lactate, %	72 (58–83)	60 (55–64)	0.659 (0.581–0.736)
SIRS, %	98 (89–100)	23 (19–27)	0.672 (0.599–0.745)
qSOFA, %	46 (32–60)	86 (83–90)	0.717 (0.673–0.758)
qSOFA + lactate, %	72 (60–85)	73 (68–77)	0.754 (0.712–0.793)
Full SOFA, %	82 (71–93)	56 (51–61)	0.795 (0.755–0.831)

*SIRS* systemic inflammatory response syndrome, *qSOFA* quick sequential organ failure assessment, *SOFA* sequential organ failure assessment, *AUROC* area under the receiver operating characteristic curve

### Predictive performance of the qSOFA + lactate score

**Fig. 1:**
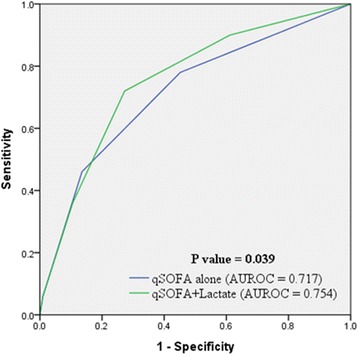
Receiver operation characteristic curve for patient mortality using qSOFA alone and qSOFA + lactate score. The combined qSOFA and lactate level score has a higher AUROC than qSOFA alone (AUROC = 0.754 vs. 0.717, *p* = 0.039, respectively). AUROC = area under receiver operating curve, qSOFA = quick sequential organ failure assessment, SOFA = sequential organ failure assessment

When the qSOFA score was combined with one additional point for hyperlactatemia, its predictive performance was significantly improved compared to that seen with the qSOFA alone (AUROC = 0.754, 95% CI = 0.712–0.793 vs. AUROC = 0.717, 95% CI = 0.673–0.758 *p* = 0.039, respectively) (Fig. 1). The qSOFA + lactate score had a higher sensitivity [72% (60–85%) vs. 46% (32–60%), respectively] with little change in specificity [73% (68–77%) vs. 86% (83–90%), respectively] (*p* < 0.001) compared with that of the qSOFA score alone.

### Comparison of the qSOFA + lactate score with the full SOFA score

**Fig. 2:**
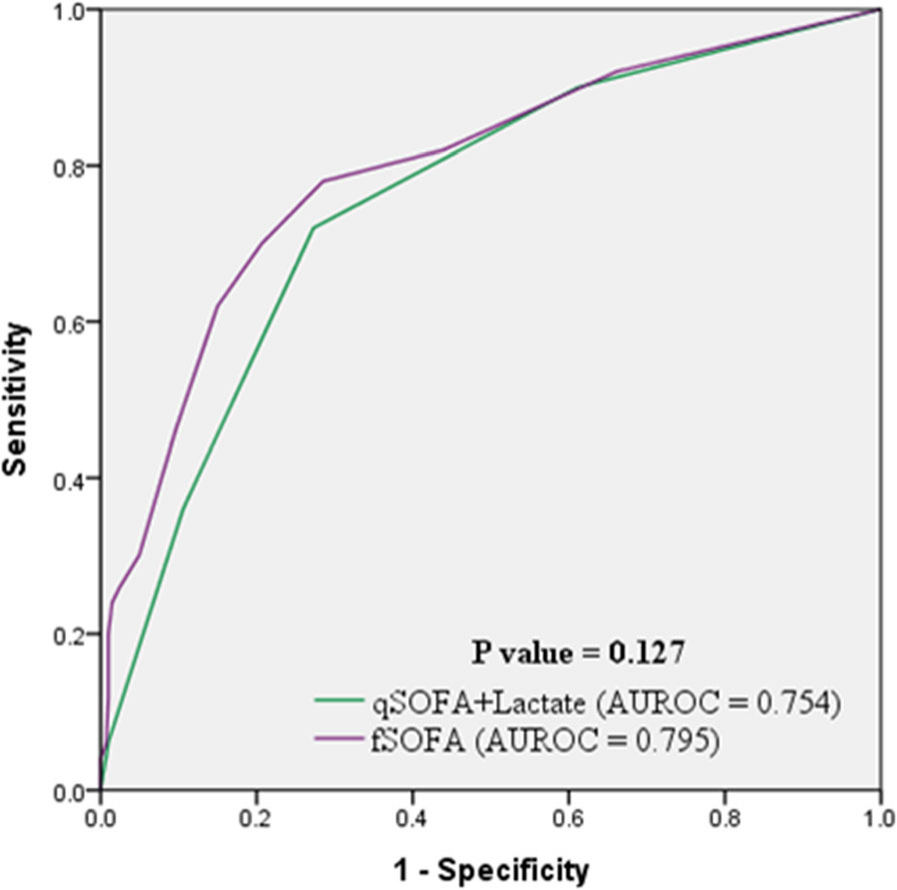
Receiver operating characteristic curve for patient mortality using qSOFA + lactate and full SOFA scores. The combined qSOFA and lactate level score has a comparable AUROC with full SOFA score (AUROC = 0.754 vs. 0.795, *p* = 0.127, respectively). AUROC = area under receiver operating curve, qSOFA = quick sequential organ failure assessment, SOFA = sequential organ failure assessment

The qSOFA + lactate score had a predictive performance comparable to the full SOFA score (AUROC = 0.754, 95% CI = 0.712–0.793 vs. AUROC = 0.795, 95% CI = 0.755–0.831, respectively; *p* = 0.127) (Fig. 2) (Table 3). The full SOFA score had a higher sensitivity [82% (71–93%) vs. 72% (60–85%)], and a lower specificity [56% (51–61%) vs. 73% (68–77%)], compared to the qSOFA + lactate score, but these differences were not statistically significant (*p* = 0.123) (Table 3).

## Discussion

Our study results showed that the qSOFA alone has a poor sensitivity and a modest predictive performance. There are rising concerns on the poor sensitivity of qSOFA, despite its high specificity, and potential delays in identification and resuscitation for patients at a high risk of mortality [16]. The addition of one point for hyperlactatemia (plasma lactate level > 2.0 mmol/L) resulted in improved sensitivity with a slightly decreased specificity. The addition of the use of serum lactate levels to the qSOFA improved the predictive performance significantly, increasing it to a level comparable to that of the full SOFA. The strengths of the qSOFA include its simplicity, rapidity, and ease of performance for all health care providers without requiring complicated equipment or laboratory results. However, after the qSOFA was introduced, we observed that surgical patients who required emergency gastrointestinal surgery rarely presented to the ED with AMS. In a study of ED patients with suspected infection conducted by Williams et al. [8], only 5.1% of patients had AMS, while 21.1% of patients had a respiratory rate ≥ 22 breaths/min, and 26.8% had a systolic blood pressure ≤ 100 mmHg. Our study also demonstrates that only 15 patients (3.3%) in our study population (457 patients) had AMS. In this population of patients, the most common presentation was abdominal pain with hypotension and tachypnea. The tachypnea was thought to be because of shallow respirations caused by diffuse peritoneal irritation in some patients. Because so few patients presented to the ED with AMS, only 17% of our patients had a qSOFA score ≥ 2, making it difficult to identify and screen patients at a high risk of mortality. In our study group, 27 patients with a qSOFA score < 2 did not survive. Other studies have emphasized the poor sensitivity of qSOFA as a screening test for sepsis [8, 9].

To improve the sensitivity of qSOFA, we analyzed several combination scores including qSOFA and diagnostic markers of inflammation, such as C-reactive protein, white blood cell count, delta neutrophil index, and plasma lactate concentration. As suggested in many studies, plasma lactate concentration is an important predictor of mortality in critically ill patients [11–13]. Plasma lactate concentration alone with a cutoff value of 2 mmol/L did not show a good predictive performance in our study, but when combined with qSOFA, it was the best predictor of in-hospital mortality in our study population.

The rapid point-of-care measurement and accuracy of plasma lactate levels is an important advantage [17]. Singer et al. reported that an early bedside point-of-care (POC) lactate evaluation in the ED was associated with the early administration of intravenous fluid resuscitation and improved mortality in patients with suspected sepsis [18]. The use of plasma lactate levels improves the discriminatory ability of qSOFA without complicating its use as a quick and simple screening method.

The association between full SOFA scores and patient mortality has been confirmed in previous studies [5, 19–21]. However, the calculation of the full SOFA score is time-consuming since many laboratory values and a significant amount of clinical information are needed. Therefore, the use of full SOFA scores is only suited for patients in an ICU environment. The use of the qSOFA + lactate score is a simple and rapid way to screen patients at high risk of mortality in pre-ICU, such as in the ED, or even in pre-hospital settings with a predictive performance comparable with that seen with the full SOFA score. The use of the qSOFA + lactate score will assist clinicians in immediately assessing the risk of mortality in patients with suspected intra-abdominal infection, allowing more rapid resuscitation and control of the infective source and reducing patient mortality.

### Strengths and limitations of our study

A strength of this study is that it is the first to be performed solely in the surgical population. Early resuscitative therapy can be delivered to patients with intra-abdominal infection who have a high risk of mortality based on the results of a simple screening using qSOFA and a plasma lactate measurement. This could lead to an improved survival rate for patients who require emergency gastrointestinal surgery for a suspected intra-abdominal infection.

The present study has several limitations. First, surgical patients presenting to the ED are likely to get individualized treatment and do not always follow a tailored protocol. In a study performed by Rivers et al. in 2001 [22], patients requiring emergent surgery were excluded from the study protocol because they had a variety of different infections and had a different degree of urgency for surgical intervention. The diversity of patient characteristics and treatments in our study may have affected the outcome of our study. Second, our study is a retrospective study performed in a single center with a small study population. Due to the retrospective nature of the study, all of our patients had already undergone emergency gastrointestinal surgery. Prospective studies of patients presenting to the ED with suspected intra-abdominal infection, regardless of a consequent emergency gastrointestinal surgery, are needed to clarify the benefits of using the qSOFA + lactate score compared to qSOFA for predicting patient mortality.

## Conclusions

The qSOFA score alone has poor sensitivity in screening surgical patients presenting to the ED with a high risk of mortality. Combining the qSOFA score with an additional one point for hyperlactatemia has a better predictive performance with higher sensitivity for patient mortality than that seen with qSOFA alone and comparable to that seen with full SOFA.

## Abbreviations

AMS: Altered mental status
AUROC: Area under receiver operating characteristic curve
AVPU: Alert/verbal/painful/unresponsive
CI: Confidence interval
ED: Emergency department
EMR: Electronic medical record
ICU: Intensive care unit
IQR: Interquartile range
POC: Point-of-care
qSOFA: Quick sequential organ failure assessment
ROC: Receiver operating characteristic
RR: Respiratory rate
SBP: Systolic blood pressure
SIRS: Systemic inflammatory response syndrome
SOFA: Sequential organ failure assessment
